# Systemising triage: COVID-19 guidelines and their underlying theories of distributive justice

**DOI:** 10.1007/s11019-022-10101-3

**Published:** 2022-07-07

**Authors:** Lukas J. Meier

**Affiliations:** Churchill College, University of Cambridge, Storey’s Way, Cambridge, CB3 0DS UK

**Keywords:** COVID-19, Corona, Triage, Prioritisation, Guidelines, Distributive justice

## Abstract

The COVID-19 pandemic has been overwhelming public health-care systems around the world. With demand exceeding the availability of medical resources in several regions, hospitals have been forced to invoke triage. To ensure that this difficult task proceeds in a fair and organised manner, governments scrambled experts to draft triage guidelines under enormous time pressure. Although there are similarities between the documents, they vary considerably in how much weight their respective authors place on the different criteria that they propose. Since most of the recommendations do not come with ethical justifications, analysing them requires that one traces back these criteria to their underlying theories of distributive justice. In the literature, COVID-19 triage has been portrayed as a value conflict solely between utilitarian and egalitarian elements. While these two accounts are indeed the main antipodes, I shall show that in fact all four classic theories of distributive justice are involved: utilitarianism, egalitarianism, libertarianism, and communitarianism. Detecting these in the documents and classifying the suggested criteria accordingly enables one to understand the balancing between the different approaches to distributive justice—which is crucial for both managing the current pandemic and in preparation for the next global health crisis.

## Introduction

In advanced economies, critical-care resources usually match or even exceed demand (Australian and New Zealand Intensive Care Society 2020, p. 4). Should local supplies become temporarily depleted, for example following a major transportation accident, patients can still be transferred to neighbouring facilities. Pandemics are unique in that they affect the whole world simultaneously. While medical staff usually deliver patient-centered care, the rationing that such a situation requires forces them also to adopt the perspective of population health. They must now balance individual needs with collective demands (Antommaria et al. 2020, p. 1; Jöbges et al. 2020, p. 1; Vergano et al. 2020, p. 1). In some developing economies, where medical resources are scarce even during normal times, capacities are exhausted yet more quickly in health crises (National Bioethics Committee of Pakistan 2020, p. 4).

When the COVID-19 pandemic began to unfold in early 2020, Italy was—following China—the second country that was hit with full force. In some regions more than ten people were competing for each ICU bed (Italian Committee for Bioethics 2020, p. 12). Overwhelmed by the rapid influx of patients, medical staff had no choice but to invoke triage even before any protocols could be drafted (Faggioni et al. 2021, p. 301; Vergano et al. 2020, p. 1). Treating physicians ‘all seemed exquisitely uncomfortable when asked to describe how these rationing decisions were being made. My questions were met with silence’, a correspondent recalls (Rosenbaum 2020, p. 1874). Seemingly, approaches to triage varied even within the same hospital.

Other countries soon found themselves in similar situations (Herreros et al. 2020, p. 455; Orfali 2020, p. 677 f.; Orfali 2021, pp. 18–20). Who should be treated first? Medical parameters provide the basis for clinical prognoses, but without clear criteria, informed by carefully balanced moral values and ethical principles, they are inadequate to guide decision-making on this unprecedented scale (Lewandowski and Schmidt 2020, p. 43; Persad et al. 2009, p. 423). Comprehensive instructions are also crucial to avoid unjust decision-making, to ensure that the best possible outcome is achieved (Jöbges et al. 2020, p. 3; Maves et al. 2020, p. 217), to provide legal clarity, and to take at least some of the enormous responsibility that health-care workers face off their shoulders (Jöbges et al. 2020, p. 10). Governments around the world therefore scrambled experts to draw up recommendations for prioritisation.

Although there are similarities between the documents, they vary considerably in some of the criteria that they propose, as several illuminating comparisons have shown (Ehni et al. 2021; Jöbges and Biller-Andorno 2020; Jöbges et al. 2020; Lewandowski and Schmidt 2020). These criteria do not come with ethical justifications, and the currently available comparisons portray the value conflicts that permeate the guidelines as tensions solely between utilitarian and egalitarian elements (Ehni et al. 2021, p. 126 f.; Jaziri and Alnahdi 2020, p. 9; Jöbges et al. 2020, p. 2 f.; Savulescu et al. 2020a, p. 10 f.). While these two accounts are certainly the main antipodes, I shall argue that in fact all four classic theories of distributive justice (Beauchamp and Childress 2013, p. 254; Meier et al. 2022, p. 11) are at work in the documents: utilitarianism, egalitarianism, libertarianism, and communitarianism. Detecting these enables one to understand the respective balancing between different values that the countries favour and to classify their stances on the issue of triage.

Triage guidelines were issued in relatively few countries (World Health Organization 2021). This analysis includes those that are of nationwide character and available in either English, French, or German. Triage policies for local application—like those in the United States—were excluded.^1^ 13 documents remained to be compared, namely the guidelines by the Australian and New Zealand Intensive Care Society (2020), the Austrian Bioethics Commission (2020), the Belgian Advisory Committee on Bioethics (2020), the British Medical Association (2021), the Canadian Medical Association (2020), the German Interdisciplinary Association for Intensive and Emergency Medicine (2020), the Department of Health of Ireland (2020), the Italian Committee for Bioethics (2020), the National Bioethics Committee of Pakistan (2020), the Critical Care Society of Southern Africa (2020), the Spanish Ministry of Health (2020), the Swedish Council on Medical Ethics (2020), and the Swiss Academy of Medical Sciences (2021).

In some of these countries, several bodies drafted recommendations simultaneously. While it would have been desirable to compare *all* available texts, their sheer number renders such an approach unfeasible. I therefore gave priority to documents officially adopted by the state or the respective nationwide specialist council. Although this means that intranational comparisons are beyond the scope of this article, I still additionally include the guidelines issued by the Italian Society of Anesthesia, Analgesia, Resuscitation and Intensive Care (2020). Since Italy was the first country in which COVID-19 triage became a reality, this very early publication was internationally the most widely discussed one (Grasselli et al. 2020; Orfali 2020; Rosenbaum 2020, p. 1874).

I shall proceed as follows. I will, as briefly as possible, introduce each theory of distributive justice before screening the guidelines for elements of the respective account. In discussing these, I shall point out weaknesses and possible objections, but remain mostly neutral towards the proposed criteria as the aim of this article is to categorise rather than to evaluate. In the end, we will then be able to locate the guidelines’ different positions on spectra between utilitarian, egalitarian, libertarian, and communitarian values.

## Utilitarian elements

Utilitarianism is the view that the morally right action is the one that results in the greatest overall good, that is, in the greatest amount of good for the greatest number of people. It is a consequentialist theory: only the *outcomes* of actions matter. An action that generates good consequences is regarded as virtuous; one that brings about bad consequences is reprehensible. Other factors, including the agent’s motivation, are irrelevant. Actions can therefore not be *intrinsically* right or wrong (Moore 2005, p. 30). There may, for instance, be circumstances in which doctors are deemed to act morally praiseworthy when they withdraw life support from one of their patients in order to save more lives overall. This is in stark contrast with deontological ethics, according to which there are certain actions that are categorically wrong and must not ever be performed—regardless of their effects.

What is a good consequence? There is much debate among consequentialists regarding which welfare function is to be maximised (Beauchamp and Childress 2013, p. 254; Savulescu et al. 2020b, p. 624). Classic utilitarians like Jeremy Bentham and John Stuart Mill regarded pleasure as the ultimate goal that morally good actions promote. Humans, argued Bentham, are governed by pleasure and pain. Making the principle of utility the basis of one’s moral theory reflects this subjection (Bentham 1789, p. i). Nowadays, consequentialist views do not constitute a unified theory but a cluster of related accounts. However, well-being is still at their heart (Savulescu et al. 2020b, p. 628).

In a triage situation, available resources are employed as efficiently as possible. Consequently, utilitarian ideals appear to be inbuilt into the very concept of triage. Many of the analysed guidelines propose allocating health care in a way that maximises the total *number of lives* saved. According to the Critical Care Society of Southern Africa (p. 4), ‘the primary goal of the allocation framework is to maximize benefit to populations of patients, specifically by maximizing survival to hospital discharge and beyond for as many patients as possible’. The Austrian Bioethics Commission (p. 12), the German Interdisciplinary Association for Intensive and Emergency Medicine (p. 4), and the Swiss Academy of Medical Sciences (p. 3) echo this criterion, all emphasising that the decisive parameter is the patient’s predicted ability to leave the ICU alive.

‘Other things being equal, we should always save five lives rather than one. However, other things are rarely equal’, remark Persad et al. (2009, p. 425).^2^ Some lives can be extended longer than others. Therefore, maximising the outcome need not refer to the total number of individual lives, but may instead mean striving for the greatest possible *amount of aggregated life time* saved with the resources available. This implies shifting the focus from short-term to long-term survival.

Using long-term survival as the decisive criterion reflects the realisation that life is valuable not merely in and of itself, but also due to the wellbeing to which it gives rise (Wilkinson 2021, p. 54). From a utilitarian perspective, it is especially important for *how long* a benefit will be enjoyed as time is a crucial multiplier in calculating the overall amount of good being generated (Savulescu et al. 2020b, p. 623). The Canadian Medical Association (p. 2) recommend this criterion alongside the aforementioned one:

Priority for limited resources should aim both at saving the most lives and at maximizing improvements in individuals’ post-treatment length of life. Saving more lives and more years of life is a consensus value across expert reports. It is consistent both with utilitarian ethical perspectives that emphasize population outcomes and with nonutilitarian views that emphasize the paramount value of each human life.

Similar goals are also articulated by the British Medical Association (p. 5), the Department of Health of Ireland (p. 17), the National Bioethics Committee of Pakistan (p. 5), and the Italian Society of Anesthesia, Analgesia, Resuscitation and Intensive Care (pp. 3, 5). The first edition of the Swiss guidelines even contained the strict instruction that patients who have a predicted life expectancy of below 12 months are to be denied admission to the ICU. This passage was removed in the current edition, however. Relying on aggregated life time would inevitably result in de-facto prioritisation of younger patients and of those with fewer comorbidities (Emanuel et al. 2020, p. 5; Jöbges et al. 2020, p. 2). This criterion is therefore less egalitarian than the total number of lives saved, which limits each patient’s claim to a maximum of one (Reid 2020, p. 528).

However, aiming for the best consequences need not translate to saving lives or even life time; it could also be interpreted as endeavouring to achieve the best aggregated *quality of life* for the greatest number of people. The Australian and New Zealand Intensive Care Society (p. 6), the Belgian Advisory Committee on Bioethics (p. 12), and the Swedish Council on Medical Ethics (p. 64) issued recommendations to this effect, albeit only as one among several other goals. The Austrian Bioethics Commission (p. 6), the Swiss Academy of Medical Sciences (p. 5), and the Canadian Medical Association (p. 2) explicitly reject this criterion—the latter on the grounds that applying it would require taking into account information that is not available in emergency situations and also be time-consuming. Allocating according to qualitative considerations can be incompatible with saving as many lives as possible. Moreover, if not applied carefully—that is, also drawing on the individual’s self-assessment rather than focusing solely on third-personal evaluation—it poses the danger of disadvantaging disabled patients (Bullinger 2014, p. 101; Perron et al. 2002, p. 563 f.; Woopen 2014, p. 142).

Finally, consequentialist principles can also dictate that factors beyond medical parameters play a role in triaging—namely, when a better overall outcome can be achieved by prioritising certain subgroups for their specialist skills. In three of the analysed guidelines, one finds this consideration in the form of recommending privileged access for clinical staff. The rationale behind this proposal is that health-care workers are essential to managing the pandemic response, so that their incapacitation is likely to entail greater losses on any of the aforementioned scales than in the case of other individuals who require admission. Consequently, one might come to the conclusion that medical staff should enjoy privileged access for their *instrumental* value, which means that, rather than deeming their lives worthier of protection, it is these professionals’ ability to save other members of society that entitles them to special treatment.

While the guidelines by both the Canadian Medical Association (p. 3) and the National Bioethics Committee of Pakistan (p. 8) contain instructions to this effect, the British Medical Association (p. 8) refer to a much larger group of people, including even individuals who maintain critical infrastructure, produce medication, or carry out administrative functions among those to be prioritised. There is, of course, great disagreement about whose tasks are indispensible in a pandemic (Persad et al. 2009, p. 426; Savulescu et al. 2020b, p. 625), with especially egalitarians being critical of priority treatment for any select groups.

## Egalitarian elements

In a sense, utilitarian views effectively promote impartiality. When the consequences of actions are calculated, all individuals are treated equally: ‘Everybody to count for one, nobody for more than one’ is, after all, the famous dictum attributed to Bentham (Mill 1863, p. 91). Historically, utilitarianism was conceived as a liberating theory for the disenfranchised classes in the society of the nineteenth century (Savulescu et al. 2020b, p. 621). However, by its very nature, triage implies prioritising certain people over others—be it for their medical parameters or their instrumental value in a health emergency (Beauchamp and Childress 2013, p. 292). As we have seen, utilitarians then prefer maximisation of aggregate utility over equal distribution. Egalitarians hold the opposite view: when the two goals are in conflict, it is most important that everyone receives an equal share—even if this comes at the cost of lower overall utility.

Egalitarianism is a set of doctrines according to which all human beings are of equal fundamental worth and moral status and should therefore treat one another as equals. Although John Locke’s writings are mainly associated with liberal ideals, it was he who formulated the egalitarian principle that all people are endowed with the same natural moral rights. These rights precede, and exist independently of, any formal agreements. They constitute claims that any person has against everyone else and that must be respected unconditionally unless forfeited or waived (Locke 2003, pp. 101–106). Treating all humans as equals is also a central demand of some religious faiths and one of the basic principles of modern constitutions. Thus, equality is widely regarded as the essence of justice (Mill 1863, p. 67).

While favouring one’s friends and family over other people may appear legitimate, it is also reasonable to suppose that more stringent egalitarian principles apply to public policies than to individual conduct. We expect of states that they behave towards their citizens according to impartial standards (Arneson 2013, sec. 7). Consequently, modern egalitarian goals include equality of income, a fair distribution of wealth within society, and equality of opportunity (Rawls 1999, p. 63).

It is no surprise, then, that egalitarian elements permeate most of the triage guidelines. There is, for example, wide agreement that *all* patients, whether suffering from COVID-19 or from other conditions, should be given equal chances of receiving treatment;^3^ and that impartiality should also be exercised with regard to other patient characteristics like disability, ethnicity, insurance status, nationality, religion, sex, social status, or wealth.^4^ As the Italian Committee for Bioethics (p. 8) highlights, one must not adopt any criterion according to which patients would be excluded ‘because they belong to a category established a priori’.

There is one parameter, however, whose application has been discussed controversially: *age*. Studies have shown a strong negative correlation between patient age and the likelihood of surviving COVID-19 infections (O’Driscoll et al. 2021). This lead some bodies to advocate age as one criterion of prioritisation—among them the National Bioethics Committee of Pakistan (p. 5) and the British Medical Association (p. 5).^5^ The recommendations issued by the Swiss Academy of Medical Sciences (p. 7) are unique in that they even set a firm age-related cut-off point (patient age > 65 years and Clinical Frailty Scale ≧ 7, or patient age > 85 years and CFS ≧ 6) beyond which patients are to be refused entry to the ICU.^6^

Several drafters explicitly reject this approach. The Department of Health of Ireland (p. 17) argues that ‘categorical exclusion e.g. on the basis of age should be avoided as this can imply that some groups are worth saving more than others and creates a perception of unfairness’.^7^ The Austrian (p. 12), German (p. 5), Spanish (p. 8), and Swedish (p. 64) guidelines contain passages to the same effect. The Australian and New Zealand Intensive Care Society (p. 5) point out that agreeing on exclusion categories that are non-discriminatory would be very difficult and that continual adjustments would be required to reflect the dynamics of the pandemic. Conversely, the British Medical Association (p. 8) opine that

indirect discrimination would be lawful in the circumstances of a serious pandemic because it would amount to ‘a proportionate means of achieving a legitimate aim’, namely saving the maximum number of lives by fulfilling the requirement to use limited NHS resources to their best effect.^8^

These opposing stances provide a vivid illustration of the tension between consequentialist and egalitarian ideals.

That this value conflict does not only exist between countries but also causes controversies on a national level is exemplified by the case of Italy: while the Italian Committee for Bioethics (p. 3) explicitly reject age as prioritisation criterium, the Italian Society of Anesthesia, Analgesia, Resuscitation and Intensive Care (p. 5) argue that an age limit for admission to the ICU may ultimately become necessary since ‘a longer and, hence, more “resource-consuming” clinical course may be anticipated in frail elderly patients with severe comorbidities, as compared to a relatively shorter, and potentially more benign course in healthy young subjects’.

A special case of age-related criterion, which has repeatedly been suggested in the literature (Harris 2001, pp. 91–94; Persad et al. 2009, p. 428; Williams 1997), found its way into only few guidelines: the so-called *fair-innings* model. Instead of setting an absolute age limit, the focus is placed on intergenerational equity. Proponents of this approach maintain that everyone should be entitled to an average life span (Williams 1997, p. 119). The Australian and New Zealand Intensive Care Society (p. 6) employ this very rationale. When patients are ranked similarly in terms of clinical priority, ‘younger patients who have lived through fewer life stages are prioritised over older patients’. Corresponding suggestions can be found in the guidelines by the Canadian Medical Association (p. 3) and the National Bioethics Committee of Pakistan (p. 8), with the latter even specifying five age ranges to be compared. The Belgian Advisory Committee on Bioethics (p. 13) and the Spanish Ministry of Health (p. 4) explicitly reject this criterion—the latter because it is taken to constitute a violation of equal human dignity. One may reply, however, that allocation based on age is less discriminating than according to other fixed parameters since *all* people who are elderly now were once younger and thus below the priority age (Persad et al. 2009, p. 429).

When neither age nor any other features that are not directly linked to the individual’s health status are supposed to play a role in prioritisation, how shall doctors decide between patients or groups of patients who are predicted to have comparable chances of survival? Prima facie, conducting a *lottery* appears to be the most egalitarian way of allocating resources because it mirrors all participants’ equal claim and dignity. Random allocation is quick and prevents marginal differences—for example in clinical scores^9^—from yielding categorical differences in treatment (Persad et al. 2009, p. 423; Reid 2020, p. 527; Savulescu et al. 2020a, p. 13; Stone 2020, p. 580). However, only the Canadian Medical Association (p. 3) advocate this means of decision-making, and it remains limited to patients with similar prognoses. The Swiss Academy of Medical Sciences (p. 5) explicitly advise against employing lotteries. What difficulties may random allocation entail?

Lotteries ‘prevent decisions from being made on the basis of reasons’ (Stone 2011, p. 16). While this pre-empts the introduction of systematic biases (Reid 2020, p. 528), giving everyone the same chance of access to treatment is not only unacceptable when there are major medical differences that suggest otherwise; it also entails the impossibility of *redressment*. The result would then not be a situation in which differences between individuals are equalised, but one in which the health-care system does not rectify interpersonal imbalances. This argument, too, is reflected in a guideline: the Austrian Bioethics Commission (p. 6) insist that even in a situation of undersupply, patients who are physically or psychologically disadvantaged should receive greater care and be allocated more resources than others to give everyone an equal chance.

Demanding resource-consuming compensation for individual vulnerabilities is, of course, in conflict with the utilitarian principle of maximising the outcome for the greatest possible number of people in circumstances of global scarcity. This might be why the Australian and New Zealand Intensive Care Society (p. 7) is the only other body that issued such a recommendation.

## Libertarian elements

While redressment is usually concerned with disadvantages that people face through no fault of their own, one might—conversely—consider de-prioritising patients for negligent or unlawful *conduct*. In the current pandemic, this could, for example, apply to individuals who contracted COVID-19 because they had attended illegal gatherings or refused to wear a mask when it was obligatory to do so. The principle of formal justice—equals should be treated equally, and unequals should be treated unequally (Beauchamp and Childress 2013, p. 250 f.)—might dictate that all people who complied with the regulations form a priority group. Such a policy has intuitive appeal, as a survey conducted among the general population of Switzerland during the first wave of the pandemic shows: 29.4% of the participants regarded de-prioritisation on the basis of patients’ prior negligence as appropriate (ethix – Lab for Innovation Ethics 2020). However, criteria of this kind come with virtually unsurmountable practical hurdles (Buyx 2008, p. 873). Isolating causal factors is difficult in medicine, and certain predispositions that lie beyond the individual’s control may be contributing to the onset or to the severity of a disease (Beauchamp and Childress 2013, p. 274 f.). It is therefore not surprising that only three of the analysed guidelines even mention this possible way of prioritising. The Italian Committee for Bioethics (p. 3) regard it as ‘ethically unacceptable’; the Belgian Advisory Committee on Bioethics (p. 13) maintain that the health-care sector is not the right place to punish people for their actions; and the Swiss Academy of Medical Sciences (p. 5) emphasise that neutrality shall also apply to the patients’ vaccination status.

By considering the implications of individual conduct, one enters the realm of the third classic theory of distributive justice: libertarianism. Libertarianism is a school of thought according to which all humans are naturally in ‘a state of perfect freedom to order their actions and dispose of their possessions’ (Locke 2003, p. 101). From this basic assumption of liberalism’s founding father John Locke, it follows that anyone who undertakes to restrict this freedom, for instance through exercising political authority, must provide good reasons for doing so. The burden of proof lies ‘with those who are against liberty; who contend for any restriction or prohibition’ (Mill 2015, p. 410).

What does individual liberty entail? Classic libertarians emphasise the connection between liberty and the possession of private property. Property, they maintain, is what enables a person to live his or her life as he or she pleases and acts as the main guarantor of a free society. Consequently, they usually opt for a free-market economy with fair competition procedures and a political system in which individual autonomy is protected from governmental power. Libertarians are therefore often in favour of a minimal state that is limited to ensuring basic rights while otherwise interfering as little as possible with its citizens’ activities (Nozick 1999, p. ix).

Mainly in response to the seminal contributions by John Rawls in the second half of the twentieth century, a new generation of libertarians began to shift their focus towards social justice: how could freedom and equality be integrated into a unified whole and economic resources be distributed in a way that facilitates both aims (Rawls 1999)? It was now taken to be the state’s task to intervene with the aim of achieving a more equal distribution of property. The large family of libertarian theories is divided by many other fracture lines, which need not concern us here. Most libertarians agree that since people follow different ways of living, each individual knows best what is good for him or her. Individual freedom and autonomous choice are therefore at the heart of libertarianism.^10^

While normally patient preferences, in conjunction with doctors’ expertise, guide medical decision-making, individual choice is naturally restricted in public-health emergencies. Once having fallen ill, a patient’s exercising of his or her autonomy becomes basically confined to its negative form: while one may not be able to request specific treatment options, one can still *reject* any. Although nowadays this important patient right is, of course, recognised in virtually all states, it is explicitly enshrined only in few guidelines. This is surprising since, unlike in times of sufficient supply, each decision to decline a certain treatment or altogether to forgo attempts at saving a patient’s life exerts an immediate influence on the individuals who have a lower priority ranking on the waiting list.

All potential means of establishing individual preferences must be taken into account, demand the German Interdisciplinary Association for Intensive and Emergency Medicine (pp. 3, 6). Given that many patients will have lost decisional capacity at the time of admission, encouraging society to specify COVID-19-related treatment preferences in advance directives is crucial, as the Canadian Medical Association (p. 3) observe. The Australian and New Zealand Intensive Care Society (p. 6) discuss the important issue of establishing the goals of care in children, while the Swiss Academy of Medical Sciences (p. 8) stress the need for periodic re-evaluation of the provided intensive-care measures to ensure that they still comply with the patient’s explicit or presumed will. The Italian Committee for Bioethics (p. 10), finally, highlight the importance of maximum transparency about the health-care system’s operation in times of crisis, so that prospective patients can take truly informed and free decisions.

There is another criterion with libertarian elements: admitting patients according to the order in which they come into contact with the health-care system. Unlike any of the factors discussed in the two preceding sections, the time at which an individual enters the queue is at least partly under his or her control. Only the British Medical Association (p. 6) and the Spanish Ministry of Health (p. 3) actively endorse this so-called *first-come-first-served* principle. Although often mentioned together (American Thoracic Society 1997, p. 1288), queuing is very different from conducting a lottery: while the drawing of lots promotes true randomness, and thus ensures equality of opportunity, the order in which prospective patients arrive at hospitals is not arbitrary but rather dependent on circumstances like patient preference, physical proximity, mobility, and available infrastructure. Some of these parameters are fixed, whereas others are contingent on decisions that individuals take regarding how they want to live—for example, whether they choose to reside in rural or urban areas. As these factors vary widely across the population, arrival time at the hospital is a rather imperfect randomiser (Reid 2020, p. 527). Therefore, queuing does not belong to the egalitarian mechanisms of distribution—but it contains libertarian elements.

The Canadian Medical Association (p. 3 f.), the National Bioethics Committee of Pakistan (p. 5), and the Swiss Academy of Medical Sciences (p. 5) explicitly reject the first-come-first-served criterion.^11^ Not only would such a principle disadvantage people who happen to become infected at a later point in time in the course of the pandemic, as the Canadian guidelines (p. 4) stress; it could, notes the Spanish Ministry of Health (p. 3), result in giving preference to patients whose condition is less severe or urgent, or to patients with unfavourable prognoses for recovery over individuals who would benefit the most. Clearly, queuing is therefore difficult to integrate with predominantly utilitarian approaches. If the number of lives saved shall be maximised despite employing a first-come-first-served policy, the only available option is additionally to invoke ex-post triage.

The issue of ex-ante and ex-post triage differs in kind from the questions of distributive justice discussed so far; for rather than acting as selection criteria themselves, the two principles only specify the temporal points at which triage decisions—according to previously established selection criteria—are to be taken. Triage can occur in two different scenarios. In the first case, the ICU has still limited space and medical staff must decide whom to admit. This is the so-called *ex-ante* triage. However, potential patients may also arrive when the ICU is already operating at full capacity. For every newly admitted individual, another patient’s ongoing treatment would then have to be terminated—the so-called *ex-post* triage.

While some authors argue that admission marks the beginning of a relationship of care and trust between patient and treating staff, which consequently warrants protection (Beauchamp and Childress 2013, p. 291; Persad et al. 2009, p. 424), the British Medical Association (p. 7), just like the Australian and New Zealand Intensive Care Society (p. 7), do not see any ‘ethically significant difference’ between refusing admission (*withholding* interventions) and discontinuing life-sustaining treatment (*withdrawing* interventions).^12^ Other guidelines, among them the Austrian (pp. 9–12), the Canadian (p. 3), the German (p. 8 f.), and the Swiss (p. 8 f.), also explicitly endorse both forms of triage.

To what extent acts and omissions are ethically similar is a complex debate in which we cannot here engage (see Emanuel et al. 2020, p. 4 f.). Especially ex-post triage also raises a host of difficult juridical questions (Lewandowski and Schmidt 2020, p. 36; Lübbe 2020, p. 435 f.). However, this form of triage comes with the important advantage of offering patients ICU-trial treatments instead of relying on the often less accurate prognostic information available at the point of hospital admission. In consequence, more lives may be saved (Savulescu et al. 2020b, p. 624 f.).

An interesting difference in wording between some of the guidelines indicates that one should distinguish between two subcategories within ex-post triage. I shall term these *comparative* and *non-comparative*. The Australian and New Zealand Intensive Care Society (p. 7), for example, specify that when it ‘becomes apparent that survival is unlikely […] it is then justifiable to consider discontinuation of intensive care therapy in order to provide support to patients who are reasonably expected [to] benefit’. Similarly, the National Bioethics Committee of Pakistan (p. 5) state that ‘ventilatory support may be withdrawn from a patient assessed to have little or no chance of survival for use to help another patient judged to have greater possibility for survival’. Thus, without regard to the newly arrived individual’s prognosis, ongoing life support may only be discontinued in agreement with *patient-centered* parameters, namely, when the already admitted patient’s health status permits doing so. The decision is non-comparative. 

In contrast, the British Medical Association (p. 4) argue that

health professionals may be obliged to withdraw treatment from some patients to enable treatment of other patients with a higher survival probability. This may involve withdrawing treatment from an individual who is stable but whose objective assessment indicates a significantly worse prognosis than that of another patient who requires the same resource.^13^

Clearly, this modus operandi renders the already admitted patient’s survival dependent on *external* factors, that is, on the health status of the people on the waiting list. In principle, this approach would therefore also license the withdrawal of treatment from patients who have a rather promising prognosis—provided only that their incoming competitors are predicted a better one. The decision is comparative.

## Communitarian elements

While libertarians stress the freedom and autonomy of the individual, communitarians emphasise our dependency on, and attachment to, social collectives. An individual, wrote Aristotle (1995, p. 4269 f.),

when isolated, is not self-sufficing; and therefore he is like a part in relation to the whole. But he who is unable to live in society, or who has no need because he is sufficient for himself, must be either a beast or a god.

Being members of families, states, nations, or religious groups, we are deeply entrenched in communities that provide for us and shape our identities and values. Social bonds, culture, and traditions give meaning and direction to our lives and are essential to our well-being and our becoming moral agents; in short: to realising human potential. Outside of society, these capacities could not develop (Taylor 1990, p. 191). Consequently, communitarians maintain, each individual is indebted to his or her community and obliged to support it. Like the other three theories of distributive justice, communitarianism is not a monolithic account but a label that denotes a range of related views. Still, communitarians are unified in holding that interests of the collective take precedence over personal goals and in regarding belongingness as more important than individual freedom.

Since in modern welfare states centrally operating health-care systems have largely replaced self-organised medical efforts at the local level, communitarian debates nowadays address the relation between individuals and the whole health community. The Department of Health of Ireland (p. 6) maintains that ‘solidarity calls for a collaborative approach to pandemics that sets aside conventional ideas of self-interest or territoriality at every level of society’. Solidarity, which the Swedish Council on Medical Ethics (p. 34) define as ‘unity between people within a group, a class, a nation or the entire world, with a readiness to provide mutual assistance’, has indeed been an important element in societies’ responses to COVID-19 (Tomasini 2021).

Which triage criteria do the guidelines put forward that fall under the broad communitarian umbrella? Individuals may be prioritised in *recognition of efforts* undertaken towards the common benefit—in this case public health. The Department of Health of Ireland (p. 7), for instance, proposes that society support ‘those who face a disproportionate burden in protecting the public good’. Like the Belgian Advisory Committee on Bioethics (p. 13), it therefore opts for a higher priority ranking for medical staff who are directly involved with patient care.

Some drafters even extend the call for priority treatment beyond front-line workers. The National Bioethics Committee of Pakistan (p. 6) recommend that close family members of medical personnel also enjoy priority; the Canadian Medical Association (p. 4) suggest that the same should apply to participants in COVID-19-related research, as a reward for their contribution—albeit only as a tiebreaker criterion; and the Australian and New Zealand Intensive Care Society (p. 7) advocate privileged access for adults with caring responsibilities if they are ranked similarly in terms of medical criteria.

As detailed in the second section, *instrumental* value may also prompt preferential treatment of subgroups. However, although utilitarian and communitarian principles may thus ultimately lead to the same triage outcome, the justifications underlying the two considerations differ markedly. Resources that are distributed according to instrumental utility are directed towards the *future* and therefore expended prospectively in the hope of exerting a positive influence—for example, ‘in order to get [specialists] back into the workforce’, as the British Medical Association (p. 8) put it.

Recognition, on the other hand, is oriented towards actions that occurred in the *past*. Here, preferential treatment is granted retrospectively in appreciation of services provided to society as a whole, which may have come with higher personal risks or other burdens (Emanuel et al. 2020, p. 4; Jöbges et al. 2020, p. 10; Persad et al. 2009, pp. 424, 426). The Canadian Medical Association (p. 3) state this quite clearly: ‘Whether health workers who need ventilators will be able to return to work is uncertain but giving them priority for ventilators recognizes their assumption of the high-risk work of saving others.’ Further consequences do not matter; the community acknowledges actions that some of its members carried out to the benefit of the collective and strives to at least partly offset the risks taken or to compensate the burdens borne. The conflict between these higher priority rankings for subgroups and the egalitarian principles laid out before should be obvious.

Besides recognition of services to the common good, preferential status can also be granted based on *need*. Of the analysed guidelines, the Swedish (p. 62) is the only one that specifically employs need as allocation criterion:

According to the principle of need and solidarity, the most seriously ill and those with the poorest quality of life should be prioritised. The more serious the illness or injury, or the poorer the quality of life as a consequence, the greater the need. At the same time, there is no need for interventions that do not improve health or quality of life.

While the final sentence puts in place a backstop against medically futile treatments, the remainder of the statement runs counter to the predominantly utilitarian approach that most other guidelines take. Sickest-first allocation often trades minor gains for large costs as it ignores post-treatment prognoses (Persad et al. 2009, p. 424). Hence, prioritising according to need would in most circumstances be inversely correlated with the greatest possible number of lives saved. However, the Swedish Council on Medical Ethics (p. 28) explicitly state that the principle of solidarity must always take precedence over the principle of cost-effectiveness. The collective provides for its weakest members, irrespective of projected outcome.

## Conclusion

Triage guidelines put forward criteria for prioritising patients that are meant to be directly applicable in clinical practice. They do not usually offer comprehensive justification for *why* a particular criterion was chosen. Making explicit the underlying ethical values and tracing them back to their corresponding theories of distributive justice was therefore the aim of this paper.

In the literature, COVID-19 triage has been portrayed as a conflict solely between utilitarian and egalitarian values (Ehni et al. 2021, p. 126 f.; Jaziri and Alnahdi 2020, p. 9; Jöbges et al. 2020, p. 2 f.; Savulescu et al. 2020a, p. 10 f.). While it is true that the main dividing line is between these two antipodes, I argued that the guidelines also comprise important libertarian and communitarian elements. Only when one exposes all four poles, one can understand how much weight each country gives to each dimension of distributive justice.

One may now connect these poles and imagine the four-dimensional space that opens up as an orthogonal coordinate system, in which one axis illustrates the conflict between utilitarian and egalitarian principles, while the other axis visualises the spectrum between communitarian and libertarian values. Whether doctors are, for example, supposed to attempt to save as many lives as possible even if this would result in the systematic exclusion of people with certain medical predispositions, or shall refrain from preselecting, notwithstanding the fact that doing so would culminate in a large number of avoidable deaths, determines—among other parameters—where a guideline is located on the utilitarianism ↔ egalitarianism axis. And whether a country employs the first-come-first-served principle or prioritises those who have acted in a way that protects the community, influences, together with other factors, a guideline’s position on the libertarianism ↔ communitarianism axis.

We began by establishing which criteria fall under the *utilitarian* umbrella, and found these to be the total number of lives saved, the aggregated life time preserved, the aggregated quality of life achieved, and priority for health-care workers on grounds of instrumental value. The British Medical Association, the Canadian Medical Association, the Italian Society of Anesthesia, Analgesia, Resuscitation and Intensive Care, the National Bioethics Committee of Pakistan, and the Critical Care Society of Southern Africa advocate particularly strong utilitarian tendencies. 

Next, we considered the *egalitarian* reflections present in the documents. We looked at age-related criteria, discussed the fair-innings argument, and examined random allocation. The Austrian Bioethics Commission, the German Interdisciplinary Association for Intensive and Emergency Medicine, the Italian Committee for Bioethics, and the Spanish Ministry of Health lean especially towards egalitarian distributions.

In the category of *libertarian* elements, we found personal conduct as a factor in prioritisation, negative patient autonomy in the form of giving up one’s place on the waiting list for someone else, and allocation according to the first-come-first-served principle. The Swiss Academy of Medical Sciences most emphatically highlight libertarian values.

Lastly, we were looking for *communitarian* traces in the guidelines. To this category belong preferential resource allocation to a subgroup of people in acknowledgement of their services to public health as well as prioritisation based on need. Especially the Australian and New Zealand Intensive Care Society, the Department of Health of Ireland, the National Bioethics Committee of Pakistan, and the Swedish Council on Medical Ethics place emphasis on communitarian elements.

The Belgian Advisory Committee on Bioethics steer a middle course and would therefore be located close to the origin of the imagined coordinate system. Although only denoting temporal parameters that do not belong to any account of justice in particular, we also briefly examined which countries endorse ex-post prioritisation and introduced a novel distinction between its comparative and non-comparative form.

It is now tempting to speculate about the motifs that ultimately lead to the countries’ different positions on the two axes: may it be the Kantian heritage with its focus on autonomy that prompted the German-speaking countries to give particular weight to patient-centered considerations? Is it not obvious that, given her utilitarian tradition, the United Kingdom would predominantly sympathise with consequentialist values? And why is it that communitarian ideals play a most prominent role in the Pakistani guidelines, while the country is simultaneously also located at the very far end of the utilitarian spectrum?

These questions are fascinating, but they exceed the sope of this paper. The rapid unfolding of the COVID-19 pandemic did not leave much room for thorough deliberations and for consulting the various stakeholders, which is a process that ordinarily precedes the drafting of documents whose content is potentially instrumental in deciding who is to live and who to die (Canadian Medical Association 2020, p. 1; Jöbges and Biller-Andorno 2020, p. 4; Lübbe 2020, p. 434; Swedish Council on Medical Ethics 2020, p. 66); nor was there any time to put into writing the various ethical background assumptions. Systemising the different criteria of prioritisation that the countries chose and tracing them back to their underlying values and theories of distributive justice is therefore vital—not only for managing the current pandemic but also in preparation for the next global health crisis.

## Footnotes

1 See Antommaria et al. (2020) for a comparison of the local triage policies in the US.

2 But see Taurek (1977, p. 303).

3 Australia & New Zealand (p. 6), Canada (p. 5), Germany (p. 4), Ireland (p. 15), Italy (Italian Society of Anesthesia, Analgesia, Resuscitation and Intensive Care, p. 5), Switzerland (p. 3), United Kingdom (p. 5).

4 Australia & New Zealand (p. 6), Austria (p. 6), Belgium (p. 13), Ireland (p. 17), Italy (Committee for Bioethics, p. 7), Sweden (p. 62), Switzerland (p. 3).

5 Age is also a major decision factor in many of the local triage guidelines in the US (Antommaria et al. 2020, p. 3).

6 The first version of the document advocated a patient age of > 85 years as *frailty-independent* exclusion criterion in the event of ex-post triage. Following public debate, however, age does no longer appear as unaccompanied criterion in the current edition (p. 7 f.).

7 For a utilitarian defense, see Savulescu et al. (2020b, p. 623).

8 See Savulescu et al. (2020a, p. 12) for a discussion of proportionality.

9 The Critical Care Society of Southern Africa (p. 5), for instance, specify that only patients with a CFS ≧ 6 should receive intensive-care treatment. For individuals with a CFS of 5, this relatively small difference means categorical exclusion, as no other parameters are taken into account.

10 Some variants of classic libertarianism even reject public health care altogether as they do not recognise any welfare rights that are based on coercive taxation.

11 The members of the Belgian Advisory Committee on Bioethics (p. 14) acknowledge that they have been unable to reach a decision on this matter.

12 See Hope et al. (2012) for a comprehensive discussion of this question.

13 This stance also reflects the majority view in a survey exploring the opinions of the general public in the UK (Wilkinson et al. 2020, p. 4).

## References

[CR1] American Thoracic Society (1997). Fair allocation of intensive care unit resources. American Journal of Respiratory and Critical Care Medicine.

[CR2] Antommaria Armand H, Gibb Tyler S, McGuire Amy L, Wolpe Paul Root, Wynia Matthew K, Applewhite Megan K, Caplan Arthur (2020). Ventilator triage policies during the COVID-19 pandemic at U.S. hospitals associated with members of the Association of Bioethics program directors. Annals of Internal Medicine.

[CR3] Aristotle. 1995. *The complete works of Aristotle*, ed. Jonathan Barnes. Princeton: Princeton University Press.

[CR4] Arneson Richard, Zalta Edward N (2013). Egalitarianism. The Stanford encyclopedia of philosophy.

[CR5] Australian and New Zealand Intensive Care Society (2020). Guiding principles for complex decision making during Pandemic COVID-19.

[CR6] Austrian Bioethics Commission (2020). Zum Umgang mit knappen Ressourcen in der Gesundheitsversorgung im Kontext der Covid-19-Pandemie: Stellungnahme der Bioethikkommission.

[CR7] Beauchamp Tom L, Childress James F (2013). Principles of biomedical ethics.

[CR8] Belgian Advisory Committee on Bioethics. 2020. *Aspects éthiques relatifs à la priorisation des soins en période de COVID-19: Recommandation du 21 décembre 2020 du Comité consultatif de Bioéthique de Belgique à laquelle adhèrent l’Ordre des médecins et le Conseil supérieur de la Santé*. Bruxelles: Comité consultatif de Bioéthique de Belgique.

[CR9] Bentham Jeremy (1789). An introduction to the principles of morals and legislation.

[CR10] British Medical Association (2021). COVID-19—Ethical issues and decision-making when demand for life-saving treatment is at capacity.

[CR11] Bullinger Monika (2014). Das Konzept der Lebensqualität in der Medizin—Entwicklung und heutiger Stellenwert. Zeitschrift Für Evidenz, Fortbildung Und Qualität Im Gesundheitswesen.

[CR12] Buyx Alena M (2008). Personal responsibility for health as a rationing criterion: Why we don’t like it and why maybe we should. Journal of Medical Ethics.

[CR13] Canadian Medical Association (2020). Framework for ethical decision making during the coronavirus pandemic.

[CR14] Critical Care Society of Southern Africa (2020). Allocation of scarce critical care resources during the COVID-19 public health emergency in South Africa.

[CR15] Department of Health of Ireland (2020). Ethical framework for decision-making in a pandemic.

[CR16] Ehni Hans-Jörg, Wiesing Urban, Ranisch Robert (2021). Saving the most lives: A comparison of European triage guidelines in the context of the COVID-19 pandemic. Bioethics.

[CR17] Emanuel Ezekiel J, Persad Govind, Upshur Ross, Thome Beatriz, Parker Michael, Glickman Aaron, Zhang Cathy, Boyle Connor, Smith Maxwell, Phillips James P (2020). Fair allocation of scarce medical resources in the time of Covid-19. New England Journal of Medicine.

[CR18] ethix – Lab for Innovation Ethics (2020). Triage-Umfrage.

[CR19] Faggioni Maurizio P, González-Melado Fermín Jesús, Pietro Maria Luisa Di (2021). National health system cuts and triage decisions during the COVID-19 pandemic in Italy and Spain: Ethical implications. Journal of Medical Ethics.

[CR20] German Interdisciplinary Association for Intensive and Emergency Medicine (2020). Entscheidungen über die Zuteilung intensivmedizinischer Ressourcen im Kontext der COVID-19-Pandemie.

[CR21] Grasselli Giacomo, Pesenti Antonio, Cecconi Maurizio (2020). Critical care utilization for the COVID-19 outbreak in Lombardy, Italy: Early experience and forecast during an emergency response. JAMA.

[CR22] Harris John (2001). The value of life.

[CR23] Herreros Benjamin, Gella Pablo, de Asua Diego Real (2020). Triage during the COVID-19 epidemic in Spain: Better and worse ethical arguments. Journal of Medical Ethics.

[CR24] Hope Tony, Mcmillan John, Hill Elaine (2012). Intensive care triage: Priority should be independent of whether patients are already receiving intensive care. Bioethics.

[CR25] Italian Committee for Bioethics (2020). COVID-19: Clinical decision-making in conditions of resource shortage and the “pandemic emergency triage” criterion.

[CR26] Italian Society of Anesthesia, Resuscitation and Intensive Care (2020). Clinical ethics recommendations for the allocation of intensive care treatments in exceptional, resource-limited circumstances.

[CR27] Jaziri Raouf, Alnahdi Saleh (2020). Choosing which COVID-19 patient to save? The ethical triage and rationing dilemma. Ethics, Medicine, and Public Health.

[CR28] Jöbges Susanne, Biller-Andorno Nikola (2020). Ethics guidelines on COVID-19 triage—An emerging international consensus. Critical Care.

[CR29] Jöbges Susanne, Vinay Rasita, Luyckx Valerie A, Biller-Andorno Nikola (2020). Recommendations on COVID-19 triage: International comparison and ethical analysis. Bioethics.

[CR30] Lewandowski Klaus, Schmidt Kurt W, Woesler Martin, Sass Hans-Martin (2020). Beatmung, Triage und Scoring—Anmerkungen zur Situation in Europa zu Beginn der COVID-19-Pandemie. Medizin und Ethik in Zeiten von Corona.

[CR31] Locke, John. 2003. *Two treatises of government and A letter concerning toleration*, ed. Ian Shapiro. New Haven: Yale University Press.

[CR32] Lübbe Weyma (2020). Orientierung in der Corona-Krise? Nicht mit Doppelbotschaften. Medizinrecht.

[CR33] Maves Ryan C, Downar James, Dichter Jeffrey R, Hick John L, Devereaux Asha, Geiling James A, Kissoon Niranjan (2020). Triage of scarce critical care resources in COVID-19: An implementation guide for regional allocation. Chest.

[CR34] Meier Lukas J, Hein Alice, Diepold Klaus, Buyx Alena (2022). Algorithms for ethical decision-making in the clinic: A proof of concept. The American Journal of Bioethics.

[CR35] Mill John S (1863). Utilitarianism.

[CR36] Mill John S, Philp Mark, Rosen Frederick (2015). On liberty, utilitarianism, and other essays. On liberty, utilitarianism, and other essays.

[CR37] Moore, George E. 2005. *Ethics*, ed. William H. Shaw. Oxford: Clarendon Press.

[CR38] National Bioethics Committee of Pakistan (2020). COVID-19 pandemic: Guidelines for ethical healthcare decision-making in Pakistan.

[CR39] Nozick Robert (1999). Anarchy, state, and Utopia.

[CR40] O’Driscoll Megan, Santos Gabriel Ribeiro Dos, Wang Lin, Cummings Derek A. T, Azman Andrew S, Paireau Juliette, Fontanet Arnaud, Cauchemez Simon, Salje Henrik (2021). Age-specific mortality and immunity patterns of SARS-CoV-2. Nature.

[CR41] Orfali Kristina (2020). What triage issues reveal: Ethics in the COVID-19 pandemic in Italy and France. Journal of Bioethical Inquiry.

[CR42] Orfali Kristina (2021). Getting to the truth: Ethics, trust, and triage in the United States versus Europe during the Covid-19 pandemic. Hastings Center Report.

[CR43] Perron Noëlle Junod, Morabia Alfredo, de Torrenté Antoine (2002). Quality of life of do-not-resuscitate (DNR) patients: How good are physicians in assessing DNR patients’ quality of life?. Swiss Medical Weekly.

[CR44] Persad Govind, Wertheimer Alan, Emanuel Ezekiel J (2009). Principles for allocation of scarce medical interventions. The Lancet.

[CR45] Rawls John (1999). A theory of justice.

[CR46] Reid Lynette (2020). Triage of critical care resources in COVID-19: A stronger role for justice. Journal of Medical Ethics.

[CR47] Rosenbaum Lisa (2020). Facing Covid-19 in Italy—Ethics, logistics, and therapeutics on the epidemic’s front line. New England Journal of Medicine.

[CR48] Savulescu Julian, Cameron James, Wilkinson Dominic (2020). Equality or utility? Ethics and law of rationing ventilators. British Journal of Anaesthesia.

[CR49] Savulescu Julian, Persson Ingmar, Wilkinson Dominic (2020). Utilitarianism and the pandemic. Bioethics.

[CR50] Spanish Ministry of Health (2020). Ministry of Health report on ethical issues in pandemic situations: SARS-CoV-2.

[CR51] Stone John R (2020). Social justice, triage, and COVID-19: Ignore life-years saved. Medical Care.

[CR52] Stone Peter (2011). The luck of the draw: The role of lotteries in decision-making.

[CR53] Swedish Council on Medical Ethics (2020). Ethical choices in a pandemic.

[CR54] Swiss Academy of Medical Sciences (2021). Triage in der Intensivmedizin bei ausserordentlicher Ressourcenknappheit (Version 4).

[CR55] Taurek John M (1977). Should the numbers count?. Philosophy & Public Affairs.

[CR56] Taylor Charles (1990). Philosophy and the human sciences: Philosophical papers 2.

[CR57] Tomasini Floris (2021). Solidarity in the time of COVID-19?. Cambridge Quarterly of Healthcare Ethics.

[CR58] Vergano Marco, Bertolini Guido, Giannini Alberto, Gristina Giuseppe R, Livigni Sergio, Mistraletti Giovanni, Riccioni Luigi, Petrini Flavia (2020). Clinical ethics recommendations for the allocation of intensive care treatments in exceptional, resource-limited circumstances: The Italian perspective during the COVID-19 epidemic. Critical Care.

[CR59] Wilkinson Dominic (2021). Frailty triage: Is rationing intensive medical treatment on the grounds of frailty ethical?. The American Journal of Bioethics.

[CR60] Wilkinson Dominic, Zohny Hazem, Kappes Andreas, Sinnott-Armstrong Walter, Savulescu Julian (2020). Which factors should be included in triage? An online survey of the attitudes of the UK general public to pandemic triage dilemmas. British Medical Journal Open.

[CR61] Williams Alan (1997). Intergenerational equity: An exploration of the “fair innings” argument. Health Economics.

[CR62] Woopen Christiane (2014). Die Bedeutung von Lebensqualität—aus ethischer Perspektive. Zeitschrift Für Evidenz, Fortbildung Und Qualität Im Gesundheitswesen.

[CR63] World Health Organization. 2021. Resources on ethics and COVID-19. https://www.who.int/teams/health-ethics-governance/diseases/covid-19/resources. Accessed 17 Nov 2021.

